# HOXD‐AS2‐STAT3 feedback loop attenuates sensitivity to temozolomide in glioblastoma

**DOI:** 10.1111/cns.14277

**Published:** 2023-06-12

**Authors:** Zuo‐Xin Zhang, Peng Ren, Yong‐Yong Cao, Ting‐Ting Wang, Guo‐Hao Huang, Yao Li, Shuo Zhou, Wei Yang, Lin Yang, Guo‐Long Liu, Yan Xiang, Yu‐Chun Pei, Qiu‐Zi Chen, Ju‐Xiang Chen, Sheng‐Qing Lv

**Affiliations:** ^1^ Department of Neurosurgery, Xinqiao Hospital Third Military Medical University (Army Medical University) Chongqing China; ^2^ School of Medicine Chongqing University Chongqing China; ^3^ Department of Neurosurgery Changhai Hospital, Second Military Medical University Shanghai China

**Keywords:** chemo‐sensitivity, glioblastoma, HOXD‐AS2, STAT3, temozolomide

## Abstract

**Aims:**

Glioblastoma multiforme (GBM) is the deadliest glioma and its resistance to temozolomide (TMZ) remains intractable. Long non‐coding RNAs (lncRNAs) play crucial roles in that and this study aimed to investigate underlying mechanism of HOXD‐AS2‐affected temozolomide sensitivity in glioblastoma.

**Methods:**

We analyzed and validated the aberrant HOXD‐AS2 expression in glioma specimens. Then we explored the function of HOXD‐AS2 in vivo and in vitro and a clinical case was also reviewed to examine our findings. We further performed mechanistic experiments to investigate the mechanism of HOXD‐AS2 in regulating TMZ sensitivity.

**Results:**

Elevated HOXD‐AS2 expression promoted progression and negatively correlated with prognosis of glioma; HOXD‐AS2 attenuated temozolomide sensitivity in vitro and in vivo; The clinical case also showed that lower HOXD‐AS2 sensitized glioblastoma to temozolomide; STAT3‐induced HOXD‐AS2 could interact with IGF2BP2 protein to form a complex and sequentially upregulate STAT3 signaling, thus forming a positive feedback loop regulating TMZ sensitivity in glioblastoma.

**Conclusion:**

Our study elucidated the crucial role of the HOXD‐AS2‐STAT3 positive feedback loop in regulating TMZ sensitivity, suggesting that this could be provided as a potential therapeutic candidate of glioblastoma.

## Graphical abstract

The lncRNA HOXD‐AS2/STAT3 feedback loop attenuates temozolomide sensitivity, indicating that this pathway may offer novel therapeutic target for treatment of glioblastoma.

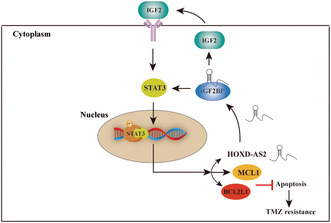

## 1 INTRODUCTION

Glioblastoma multiforme is the most devastating brain malignancy in adults.^1^ Standard treatment mainly includes surgical resection, followed radio‐chemotherapy and sequential temozolomide chemotherapy,^2^ however, the median survival is approximately 14.6 months.^3^

Temozolomide is an alkylating agent which can penetrate the blood–brain barrier and alkylate the genomic DNA at the N^7^ and O^6^ sites of guanine,^4^ inducing DNA damage and cell death. TMZ‐induced DNA damage was identified by DNA repair systems, which could affect the sensitivity to cytotoxic therapeutic agents.^5^ Although the Stupp's Protocol helped to improve prognosis of newly diagnosed GBM remarkably,^6^ the TMZ sensitivity determines the therapeutic effect and outcomes and a majority of patients do not respond to temozolomide.^7^

Long non‐coding RNAs are transcripts longer than 200 nucleotides with no protein‐coding function and involved in various cancers including glioma,^8^ affecting the cellular processes by regulating gene expression at transcriptional, post‐transcriptional and epigenetic levels.^9^ Evidences confirmed that lncRNAs play pivotal roles in regulating tumorigenesis and drug resistance of gliomas.^10^, ^11^, ^12^, ^13^ HOXD‐AS2 (HOXD cluster antisense RNA 2) is a lncRNA transcript in HOX (homeobox) gene locus and growing studies reported that HOXD‐AS2 exerts its functions in glioma progression. For instance, Peng et al reported that HOXD‐AS2 can activate cell cycle to promote glioma progression^14^; HOXD‐AS2 can also promote glioblastoma cell proliferation, migration, and invasion by regulating the miR‐3681‐5p/MALT1 signaling pathway^15^, ^16^; You et al found that TGF‐β1‐induced HOXD‐AS2 can competitively bind to KSRP to regulate MGMT expression and TMZ resistance.^17^ Therefore, underlying mechanisms of HOXD‐AS2 affecting the malignant behaviors of glioblastoma deserve an in‐depth investigation.

Signal transducer and activator of transcription 3 (STAT3) is an important transcription factor that regulates diverse cellular processes such as proliferation, apoptosis, angiogenesis, inflammation, and immune responses.^18^ Aberrant STAT3 activation occurs in various carcinomas including glioblastoma,^19^ and STAT3 signaling pathway is recognized as a main mediator hub of aberrant cellular signaling regulatory networks and leads to poor prognosis.^20^ Additionally, STAT3 inhibition could sensitize glioblastoma to temozolomide,^21^ thus STAT3 is greatly required for elucidating mechanisms of temozolomide resistance.

In our study, we found and described a HOXD‐AS2‐STAT3 positive feedback loop which attenuated temozolomide sensitivity in glioblastoma, indicating that this loop might be targeted as a novel therapeutic strategy for glioblastoma.

## 2 METHODS

### 2.1 Data and sample collection

RNA‐sequencing data of glioma samples were downloaded from The Cancer Genome Atlas (TCGA) database (https://portal.gdc.cancer.gov/) and the Chinese Glioma Genome Atlas (CGGA) database (http://www.cgga.org.cn). The MGMT (O^6^‐methylguanine‐DNA methyltransferase) promoter methylation statuses of glioblastomas in TCGA database were annotated according to previous study.^22^ Differentially expressed lncRNAs between glioblastoma and normal brain tissues were identified using “edge R” package of R software (|Log_2_FoldChange| ≥ 2, FDR < 0.01); High‐grade glioma (HGG) specimens (XQ‐HGG cohort) were obtained from patients who underwent surgery at the Department of Neurosurgery, Xinqiao Hospital of the Third Military Medical University. These specimens consisted of five adjacent‐tumor tissues, 15 grade III, and 27 grade IV glioma tissues. This study was approved by the Ethics Committee of the Xinqiao Hospital of Third Military Medical University, and written informed consents were obtained from all patients or his/her guardians.

### 2.2 Cell culture and reagents

Human U87, U251, SNB19, and LN229 glioma cells, normal glial cell HEB were obtained from ATCC (American Type Culture Collection, passages 5–20) and cultured in DMEM (Dulbecco's Modified Eagle's Medium‐High Glucose) medium supplemented with 10% fetal bovine serum under 37°C in a humidified atmosphere with 5% CO_2_. Cell lines were tested using the ATCC cell line authentication and routinely tested for mycoplasma.

Lenti‐virus (Genechem, Shanghai, China) and targeted siRNAs (GenePharma) were transfected into cells according to manufacturer's instructions; The shRNA target and siRNA sequences are listed in Table S1. Temozolomide reagent was purchased from Selleck; S3I‐201 was purchased from Beyotime Biotechnology; IL6 and IGF2 recombinant protein were purchased from MCE.

### 2.3 Bio‐informatics analysis

Nomograms were constructed with multivariate cox regression method using “rms” R package; GO (Gene Ontology) and KEGG (Kyoto Encyclopedia of Genes and Genomes) pathway analysis was performed using “clusterProfiler” R package; Gene Set Enrichment Analysis was performed using GSEA software; Gene Set Variation Analysis was performed using “GSVA” R package and FDR < 0.25 and *p* < 0.05 were considered statistically significant. Online resources of StarBase (https://starbase.sysu.edu.cn/index.php), RBPsuite (http://www.csbio.sjtu.edu.cn/bioinf/RBPsuite/), and CatRapid (http://s.tartaglialab.com/page/catrapid_group) were utilized to predict the potential proteins that interacted with HOXD‐AS2; JASPAR (https://jaspar.genereg.net/) online website was utilized to predict binding sites of STAT3 on HOXD‐AS2 promoter region.

### 2.4 RT‐qPCR assay

Total RNAs were extracted and reversely transcribed into cDNA using HiScript III RT SuperMix for qPCR Kit. The primers sequences are listed in Table S2. The relative mRNA expression was calculated by the $2^{-\Delta \Delta C_{t}}$ method.

### 2.5 Fluorescence in situ hybridization (FISH)

The FISH Probe Mix of HOXD‐AS2 and internal control probes were designed and purchased from RiboBio Co., Ltd. FISH assay was performed according to instructions of Fluorescent In Situ Hybridization Kit (RiboBio). Briefly, cells were seeded, fixed, and permeabilized; Pre‐hybridization blocking was performed at 37°C for 30 min, and the cells were hybridized at 37°C overnight in darkness. Then cells were washed by saline sodium citrate (SSC) buffer at 42°C. Subsequently, the cell nucleus was stained with 4′,6‐diamidino‐2‐phenylindole (DAPI) staining solution and then rinsed with phosphate buffered saline (PBS) buffer.

### 2.6 Sub‐cellular fractionation analysis

Sub‐cellular fractionation analysis was performed using RNA sub‐cellular Isolation Kit (ActiveMotif) according to manufacturer's instruction. 1 × 10^6^ cells were harvested and lysed; Then the lysate was centrifuged and separated into cytoplasmic and nuclear fractions; The elution was reversely transcribed into cDNA and analyzed with RT‐qPCR method and the levels of HOXD‐AS2, GAPDH (cytoplasm control), and U6 (nucleus control) were measured, respectively.

### 2.7 Cell viability assays

Cell Counting Kit‐8 assay (MCE) was performed to measure the relative cell viability after TMZ treatment for various time periods. For cell viability assay, 5 × 10^3^ cells/well were seeded in a 96‐well plate and cultured overnight. The cells were treated with TMZ at different concentrations and cultured for 72 h then the cell viability was measured immediately; For another, cells were also treated with TMZ at a concentration of 200 μM and cultured continuously and the relative cell viability was measured for various time periods. Untreated cells were used as a negative control, and each test was repeated in multiple wells.

### 2.8 Intracranial glioma model construction

The animal experiments were approved by the Laboratory Animal Welfare and Ethics Committee of Third Military Medical University and followed guidelines for animal welfare. Four‐week‐old female BALB/c nude mice were purchased from Beijing Vital River Laboratory Animal Technology Co., Ltd. U87 cells were transfected with luciferase lenti‐virus (Genepharma) alone or co‐transfected with HOXD‐AS2 shRNA lenti‐virus (or control lenti‐virus). 5 × 10^5^ cells (in 5 μL PBS) were injected into the intracranial striatum of nude mice stereotactically. The injection point was positioned 1 mm anterior and 2 mm lateral to the bregma, 3 mm below the skull bone. The mice were randomly divided into three groups: four mice in control group, five mice in TMZ treatment group, and five mice in co‐treatment group (HOXD‐AS2 knockdown + TMZ). Temozolomide (5 mg/kg, i.p.) was given to TMZ and co‐treatment groups respectively on day 7 on a 5 days on/2 days off regimen for two cycles. Mice of control group were treated with an equal volume of DMSO alone. On the day 7 and 14 after implantation, mice were anesthetized and injected intraperitoneally with luciferin (150 mg/kg; Promega), and intracranial tumors were measured with bioluminescent imaging using living imaging system. Survival analysis was performed and mice were sacrificed by euthanasia, then brain samples were taken for hematoxylin and eosin (HE) staining.

### 2.9 Immunohistochemistry (IHC) and hematoxylin–eosin (HE) staining

Mouse brain tissues were fixed, embedded, and sectioned into slides. For IHC analysis, slides were dewaxed and rehydrated. Antigen retrieval was performed using sodium citrate buffer. Slides were incubated in 3% H_2_O_2_ to block endogenous peroxidases and blocked with serum. Subsequently, slides were incubated at 4°C overnight with primary antibody against γ‐H2AX (phospho Ser139, 1:1000 dilution; Cell Signaling Technology), then incubated with a secondary antibody and then stained with diaminobenzidine, hematoxylin, and mounted; For HE staining, the slides were dewaxed and rehydrated before the nuclei were stained with hematoxylin; Sections were then rinsed and stained with eosin before being dehydrated and mounted.

### 2.10 Western blotting assay

Protein lysates were loading and separated by SDS‐PAGE gel and transferred to polyvinylidene fluoride membranes (Millipore) and then incubated with primary antibodies against STAT3 (1:1000 dilution; ProteinTech), p‐STAT3 (phospho tyrosine 705, 1:1000 dilution; BOSTER), IGF2BP2 (1:1000 dilution; ProteinTech), BCL2L1 (1:1000 dilution; BOSTER), and GAPDH (1:1000 dilution; Zsbio) overnight at 4°C, followed by incubation with the HRP‐conjugated secondary antibodies. Antigen–antibody binding signal was detected with the chemiluminescent substrates.

### 2.11 RNA‐protein pull‐down assay

1 × 10^7^ cells were lysed in IP lysis buffer (Thermo Scientific) containing protease and phosphatase inhibitor cocktail and RNase inhibitor. The HOXD‐AS2 cDNA strands were synthesized by Genepharma company. In vitro transcription with the HiScribe™ T7 High Yield RNA Synthesis Kit (NEB) was performed and biotin‐labeled HOXD‐AS2 was prepared with the Pierce RNA 3′ End Desthiobiotinylation Kit (Thermo Scientific); RNA pull‐down assay was performed using the Magnetic RNA‐protein Pull‐Down Kit (Thermo Scientific) according to manufacturer's instruction. The elution was analyzed by mass spectrometry (GeneCreate Biological Engineering Co. LTD).

### 2.12 RNA immunoprecipitation (RIP) assay

RIP assays were conducted using the Magna RIP RNA‐Binding Protein Immunoprecipitation Kit (Millipore) according to manufacturer's instruction. 1 × 10^7^ cells were lysed with lysis buffer. The beads and antibody were then rotating co‐incubated with immunoprecipitation buffer and cell lysate at 4°C overnight. The RNA‐protein complexes were isolated and digested by proteinase K, and RNAs were precipitated for cDNA synthesis and measured by real‐time PCR. The RIP primers are listed in Table S2.

### 2.13 RNA‐protein co‐localization analysis

Cells were seeded, fixed, permeabilized, and pre‐hybridized as described previously. Subsequently, hybridization was performed at 37°C in darkness overnight. Then the cells were incubated with BSA blocking buffer (PBST containing 5% bovine serum albumin) for 30 min at room temperature, and with primary antibody against IGF2BP2 (1:100 dilution; ProteinTech) for 1 h at room temperature, finally with Alexa Fluor‐488 conjugated secondary antibody and DAPI. Slides were mounted with antifade mounting medium and immediately imaged on an Olympus microscope.

### 2.14 Chromatin immunoprecipitation (ChIP) assay

Chromatin immunoprecipitation assay was performed following the manufacturer's instructions of EZ‐Magna ChIP™ A/G One‐Day Chromatin Immunoprecipitation Kit (Milipore). Briefly, 5 × 10^6^ cells were cross‐linked, quenched, washed, and then scraped and centrifuged. The cells were lysed and sonicated to shear DNA to lengths between 200 and 1000 bp. The chromatin of the lysate was incubated overnight with anti‐STAT3 antibody (ProteinTech) or normal IgG. Beads were washed and PCR was then performed. The specific primer is listed in Table S2.

### 2.15 The differentially HOXD‐AS2 expression of all foci in a clinical case with multiple GBM

A 47‐year‐old male diagnosed as multiple intracranial space‐occupying lesions (four foci) underwent surgery and pathological examination confirmed multiple glioblastoma. This patient received post‐operational concurrent radio‐chemotherapy and conventional TMZ chemotherapy. The follow‐up magnetic resonance imaging (MRI) revealed that the tumor recurrence of different regions and the HOXD‐AS2 expression values of these tumor foci were also analyzed.

### 2.16 Statistical analysis

GraphPad Prism 6.01 software was used for statistical analyses and data distributions were evaluated using a Shapiro–Wilk test. All data were presented as mean ± standard error. The Student's *t*‐test or Mann–Whitney test were used to compare two groups for continuous variables with normal distribution or non‐normal distribution; One‐way parametric analysis of variance (ANOVA) or non‐parametric Kruskal–Wallis test was used for normally or non‐normally distributed variables among at least three groups; Kaplan–Meier curves were used to describe survival associations and log rank test was used for analyzing significant differences; Pearson or Spearman correlation analysis was used to evaluate the correlations between continuous variables. All tests were two‐tailed and *p* values <0.05 were considered to be statistically significant.

## 3 RESULTS

### 3.1 Elevated HOXD‐AS2 associates with poor prognosis in glioma

**FIGURE 1:**
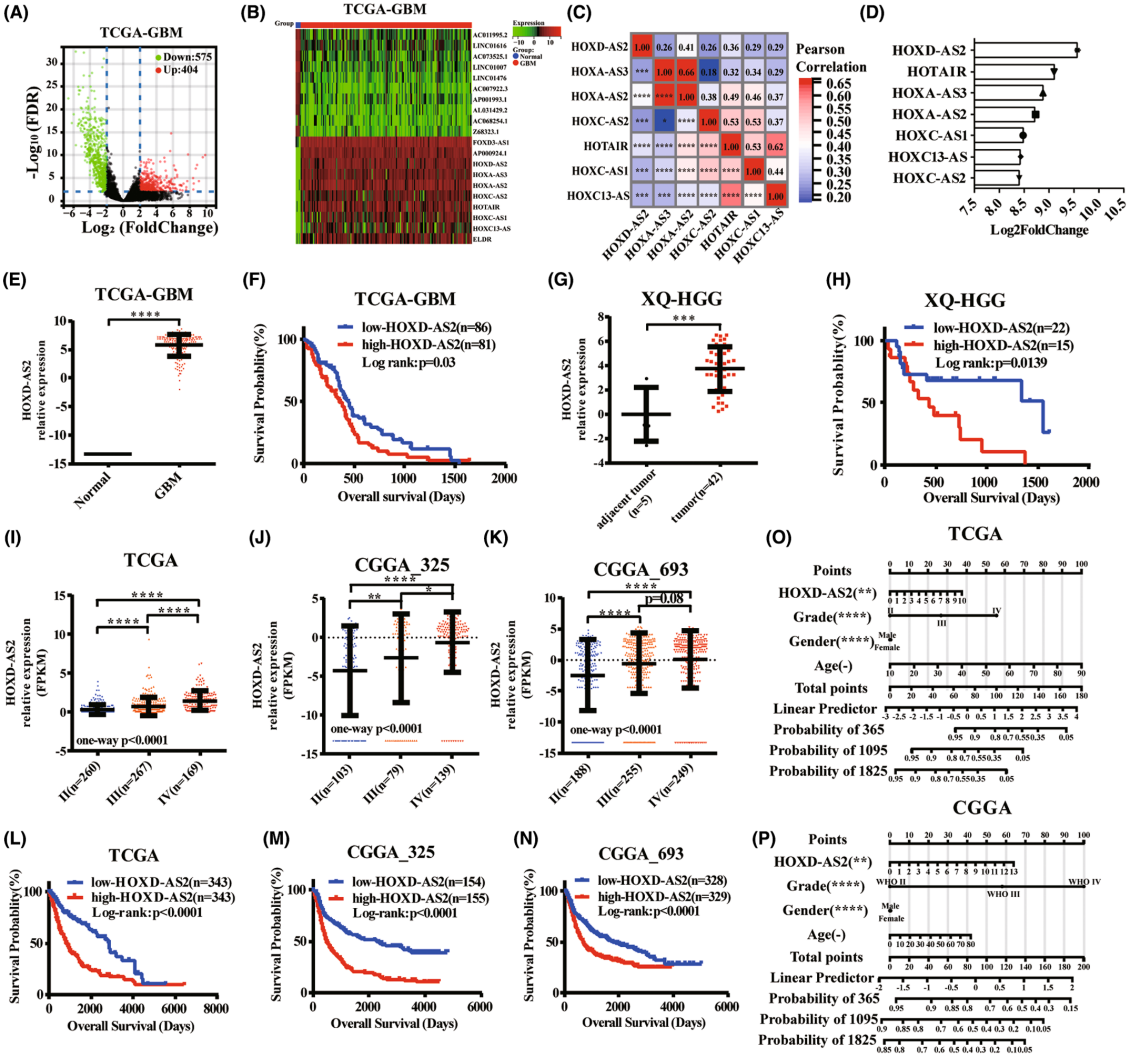
Elevated HOXD‐AS2 associates with poor prognosis and promises to be a prognostic factor in glioma. (A) Significant differentially expressed lncRNAs in glioblastoma of TCGA database (upregulated: 404, downregulated: 575); (B) Top 10 significant up/downregulated lncRNAs in glioblastoma of TCGA database; (C) The correlation of these HOX‐lncRNAs' expression (Pearson's coefficient); (D) HOXD‐AS2 was the highest upregulated HOX‐lncRNAs in glioblastoma of TCGA database; (E) The level of HOXD‐AS2 was analyzed in non‐tumor brain and glioblastoma in TCGA database; (F) A Kaplan–Meier survival curve was used to examine the relationship of HOXD‐AS2 expression and overall survival of patients with GBM; (G) The expression of HOXD‐AS2 was detected in high‐grade glioma tissues in XQ‐HGG cohort (adjacent tumor: *n* = 5; tumor: *n* = 42) by RT‐qPCR; (H) A Kaplan–Meier survival curve was used to examine the relationship of HOXD‐AS2 expression and overall survival of patients with high‐grade gliomas in XQ‐HGG cohort; (I–K) The level of HOXD‐AS2 was analyzed in gliomas of different grades in TCGA and CGGA database; (L–N) A Kaplan–Meier survival curve was used to examine the relationship of HOXD‐AS2 expression and overall survival of patients with all gliomas in TCGA and CGGA database; (O, P) Nomograms for predicting 1‐, 3‐, and 5‐year survival probability indicated that HOXD‐AS2 could be a prognostic factor in glioma in TCGA and CGGA database; Mann–Whitney test and Kruskal–Wallis test for group comparisons; Log rank test for survival analysis; The asterisks denote significance (*p < 0.05, **p < 0.01, ***p < 0.005, and ****p < 0.001).

We identified 404 upregulated and 575 downregulated lncRNAs in glioblastoma from TCGA database (Figure 1A); The heatmap illustrated the significant top 10 up/downregulated lncRNAs in glioblastoma (Figure 1B) and it attracted our attention that HOX gene related lncRNAs accounted for a larger proportion of upregulated lncRNAs (7 of 10). HOX genes are vital transcription factors that regulate cellular fate and development,^23^ additionally, the HOX‐lncRNAs have also been reported to be associated with glioma development.^24^, ^25^, ^26^ The heatmap demonstrated the significant correlations between these HOX‐lncRNAs' expressions (Figure 1C) and HOXD‐AS2 was found to be the highest upregulated in glioblastoma (Log_2_FoldChange = 9.56, Figure 1D); HOXD‐AS2 was significantly upregulated in glioblastoma and negatively correlated with overall survival (OS) of GBM patients in TCGA database (Figure 1E,F) and we validated the HOXD‐AS2 expression and its association with prognosis with the high‐grade glioma specimens we collected (XQ‐HGG cohort, Figure 1G,H); Furthermore, we investigated HOXD‐AS2 expression in glioma tissues of different pathological grades. In TCGA database, differences were observed between low‐grade (WHO II) and high‐grade glioma (WHO III, IV) mutually and Kaplan–Meier curves revealed that patients of higher HOXD‐AS2 expression experienced worse overall survival (Figure 1I,L); Similarly, in CGGA databases, we validated our results in glioma tissues of different pathological grades (Figure 1J,K,M,N); Meanwhile, the nomograms were constructed by multivariate cox regression method with HOXD‐AS2 expression value, survival time, vital status, and other clinical characteristics (age, gender, and grade) to predict the 1‐, 3‐, and 5‐year survival probability and these results indicated that HOXD‐AS2 could be a prognostic indicator. (Figure 1O,P). Our results indicated that elevated HOXD‐AS2 was associated with poor prognosis in glioma.

### 3.2 HOXD‐AS2 is involved in TMZ sensitivity regulation in glioma

**FIGURE 2:**
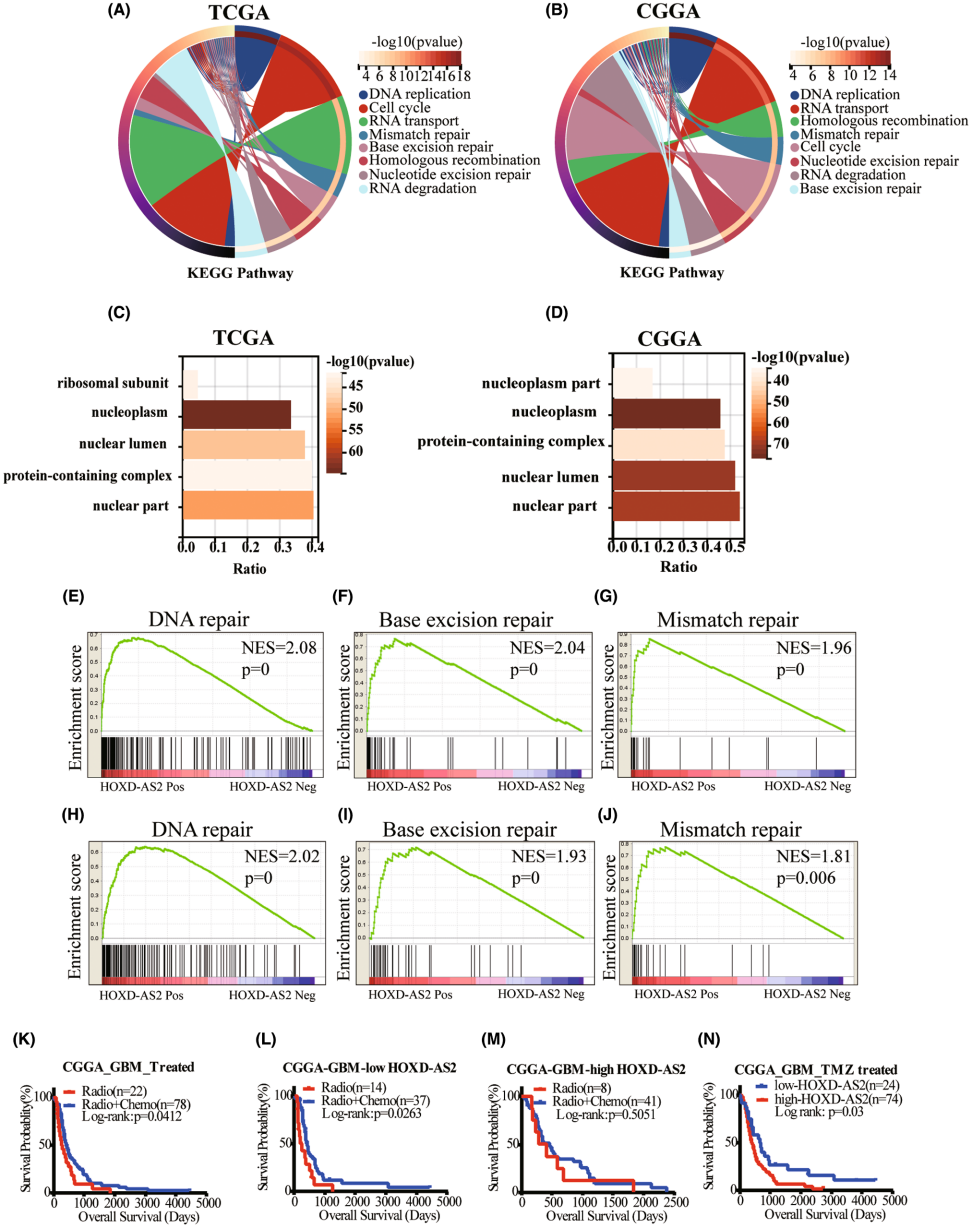
Bio‐informatic analysis reveals that HOXD‐AS2 is involved in TMZ sensitivity. (A, B) KEGG pathway analysis was performed using the HOXD‐AS2‐correlated genes and showed the functional pathways that HOXD‐AS2 involved; (C, D) GO enrichment analysis was performed using the HOXD‐AS2‐correlated genes and showed that these genes were mainly located in nucleus; (E–G) GSEA analysis was performed and demonstrated that HOXD‐AS2 was positively enriched in DNA repair, base excision repair, and mismatch repair process in TCGA and (H–J) CGGA database; (K) In CGGA cohort, patients received concurrent radio‐chemotherapy benefited better than those received radiotherapy alone; (L) In low HOXD‐AS2 group, patients who accepted chemotherapy achieved longer overall survival than the patients who did not accept; (M) In high HOXD‐AS2 group, the overall survival of patients who accepted chemotherapy was not significantly longer than those who did not accept; (N) TMZ‐treated GBM patients of low HOXD‐AS2 expression achieved longer overall survival than those of high HOXD‐AS2 expression. Log rank test for survival analysis.

Bio‐informatic analysis was performed to explore potential functions of HOXD‐AS2 in glioma. First, we identified protein‐coding genes that were significantly correlated with HOXD‐AS2 in public databases (Pearson coefficient, |*r*| ≥ 0.3, *p* ≤ 0.01). The KEGG pathway enrichment results showed that these genes were mainly involved in DNA replication, DNA damage repair, homologous recombination, and so on (Figure 2A,B); The GO enrichment results showed that these genes were mainly located in nucleus (Figure 2C,D); Furthermore, GSEA analysis consistently demonstrated that HOXD‐AS2 was positively enriched in DNA repair, base excision repair, and mismatch repair process (Figure 2E–J). TMZ‐induced DNA damage always activates repair pathways such as base excision repair, MGMT repair, and mismatch repair,^27^ which lead to chemo‐resistance. Our results indicated that HOXD‐AS2 might affect cellular response to DNA damage caused by therapeutic agents, consequently, it might regulate temozolomide sensitivity in glioma.

In CGGA cohort, the GBM patients received concurrent radio‐chemotherapy benefited better than those who received radiotherapy alone (Figure 2K), which had been a consensus, then an interesting phenomenon attracted our attention. The median expression value was set as a cut‐off to discriminate low and high HOXD‐AS2 groups, and we further divided them into TMZ‐treated and non‐TMZ‐treated subgroups; We found that in low HOXD‐AS2 group, patients who accepted chemotherapy achieved longer overall survival than patients who did not received (log rank test, *p* = 0.0263, Figure 2L); However, the same phenomenon was not observed in high HOXD‐AS2 group (log rank test, *p* = 0.5051, Figure 2M); Besides, TMZ‐treated GBM patients with low HOXD‐AS2 expression achieved longer overall survival than the patients with high HOXD‐AS2 expression (log rank test, *p* = 0.03, Figure 2N). Above all, these results indicated that low HOXD‐AS2 might sensitize GBM to temozolomide.

### 3.3 HOXD‐AS2 is a cytoplasmic lncRNA in glioblastoma

**FIGURE 3:**
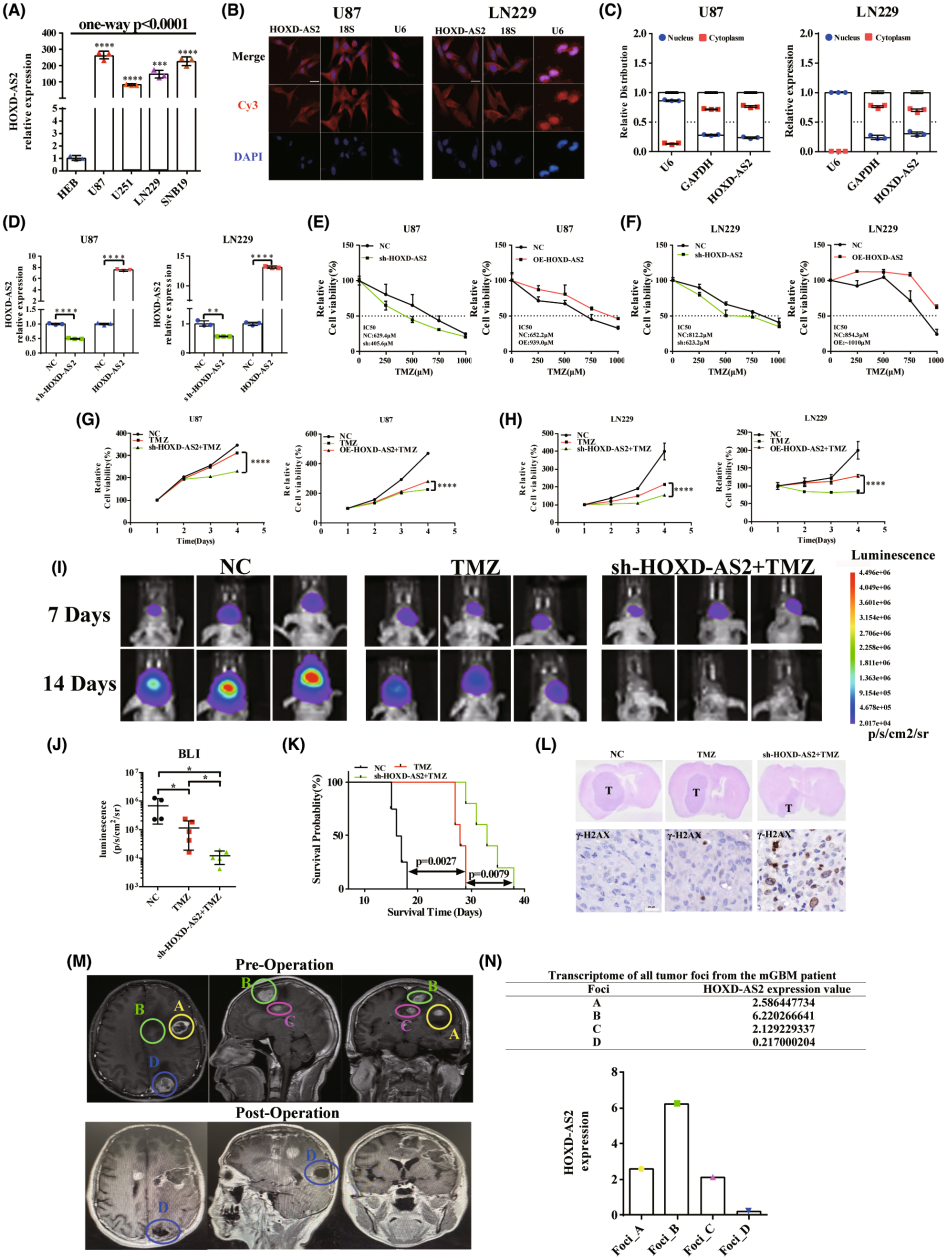
HOXD‐AS2 attenuates temozolomide sensitivity in vitro and vivo. (A) Expression of HOXD‐AS2 was determined in glioma and human glial cell lines by RT‐qPCR; (B) The sub‐cellular localization of HOXD‐AS2 in GBM cells was determined by FISH assays; (C) Sub‐cellular fractionation analysis confirmed the sub‐cellular localization of HOXD‐AS2 in GBM cells (*n* = 3 in each group); (D) Lenti‐virus were transfected into cells and the transfection efficiency was detected by RT‐qPCR method; (E) HOXD‐AS2 knockdown/overexpressed U87 and (F) LN229 cells were treated with TMZ at different concentrations for consecutive 72 h, the relative cell viability was assessed immediately by CCK8 assays; (G) HOXD‐AS2 knockdown/overexpressed U87 and (H) LN229 cells were treated with TMZ at a constant concentration of 200 μM for different time periods, the relative cell viability was assessed by CCK8 assays; (I) Representative bioluminescent images of mice intracranial gliomas in different groups on day 7 and 14 after tumor implantation (*n* = 3); (J) Bioluminescent intensity of intracranial gliomas in NC (*n* = 4), TMZ (*n* = 5), shHOXD‐AS2 + TMZ (*n* = 5) groups on day 14 after implantation; (K) Survival rate of the glioma‐bearing mice of different groups; (L) Representative HE staining images, and IHC staining images for γ‐H2AX of the mice brain sections in different groups (The tumor regions were marked by “T”); (M, N) A clinical case of multiple glioblastoma indicated that low HOXD‐AS2 had a tendency of TMZ sensitivity; The follow‐up MRI showed that the tumor foci (labeled A, B, C) manifested obvious recurrences, whereas, the foci D manifested no recurrent neoplasm; The transcriptome of the four foci showed the diverse expression of HOXD‐AS2. The experiment was repeated three times; Student's *t*‐test for group comparisons; Log rank test for survival analysis; The asterisks denote significance (*p < 0.05, **p < 0.01, and ***p < 0.005), scale bar = 20 μm.

The RT‐qPCR assays revealed that HOXD‐AS2 was remarkably upregulated in glioma cells (Figure 3A). Furthermore, we investigated its sub‐cellular localization in U87 and LN229 cell lines using FISH and sub‐cellular fractionation analysis; With reference to the corresponding indicators of GAPDH (cytoplasm), 18S RNA (cytoplasm), and U6 (nucleus), HOXD‐AS2 was predominantly localized in cytoplasm of glioblastoma cells (Figure 3B,C).

### 3.4 HOXD‐AS2 attenuates temozolomide sensitivity in vitro

Gene gain and loss function assays were performed in U87 and LN229 cells to verify previous analysis results. Cells were transfected with HOXD‐AS2 knockdown and over‐expression lenti‐virus and RT‐qPCR method was utilized to measure the transfection efficiency (Figure 3D). Lenti‐virus transfected cells were treated with several concentrations of TMZ for 72 h, then the relative cell viability and dose–response analysis was measured and analyzed. As shown in Figure 3E,F, in U87 cells, the presence of HOXD‐AS2 knocking down effectively restored sensitivity to TMZ, and the IC50 value decreased to 405.6 μM from 629.4 μM compared with the NC group; Conversely, the HOXD‐AS2‐overexpressing U87 cells exhibited resistance to TMZ, with IC50 increasing to 939.0 μM from 652.2 μM compared with the NC group; Similarly, HOXD‐AS2 silencing effectively sensitize LN229 cells to TMZ, and the IC50 value decreased to 623.2 μM from 812.2 μM compared with the NC group; Conversely, HOXD‐AS2‐overexpressed cells revealed resistance to TMZ, with IC50 increasing to more than 1010 μM from 854.3 μM compared with the NC group.

For another, cells were treated with a constant concentration of 200 μM and relative cell viability was measured for various time periods to assess the proliferation. As shown in Figure 3G,H, HOXD‐AS2 silencing combined with TMZ manifested more effective inhibition of cell proliferation than TMZ‐treated group; Conversely, HOXD‐AS2 over‐expression combined with TMZ manifested less effective growth inhibition than TMZ‐treated group. These results indicated that HOXD‐AS2 attenuated sensitivity to temozolomide in vitro.

### 3.5 HOXD‐AS2 attenuates temozolomide sensitivity in vivo

The U87 cells expressing bioluminescent reporter luciferase were injected into mice brains stereologically and in vivo orthotopic glioma models were constructed. The growth of intracranial tumors was quantified by bioluminescent intensity (BLI) using living imaging system. BLI of TMZ‐treated group was significantly lower than that of control group (Figure 3I,J), additionally, the BLI of combination treatment group was significantly lower than TMZ‐treated group (Figure 3I,J). These results indicated that combination of TMZ and HOXD‐AS2 silencing significantly inhibited the growth of intracranial tumors, leading to a significant increase in survival (Figure 3K). HE staining of mice brain sections also confirmed that combination of TMZ and HOXD‐AS2 knockdown significantly inhibited the tumor growth (Figure 3L), which was consistent with previous bioluminescent imaging. IHC staining revealed that increased γ‐H2AX (Ser139) staining was simultaneously observed in combination treatment group compared with TMZ‐treated group (Figure 3L), indicating that combination of TMZ and HOXD‐AS2 knockdown induced accumulation of DNA double‐strand breaks,^28^ which meant an increased drug‐sensitivity tendency. Our results demonstrated that HOXD‐AS2 attenuated the temozolomide sensitivity of glioblastoma in vivo.

### 3.6 Low HOXD‐AS2 had a tendency of TMZ sensitivity in clinic

Based on our previous study which reported the multi‐omics features of multi‐focal glioblastoma,^29^ we accidentally found an interesting clinical case. As shown in Figure 3M,N, the multiple GBM samples (labeled A, B, C, and D) from the same patient were analyzed by bulk transcriptome sequencing previously,^29^ which showed diverse HOXD‐AS2 expression. This patient received surgical resection and standard TMZ therapy and long‐term follow‐up was conducted. Eventually, the follow‐up MRI revealed tumor recurrence. Interestingly, we found that the tumor foci with the lowest HOXD‐AS2 expression (Foci D) showed no recurrent neoplasm, whereas the tumor foci with higher HOXD‐AS2 expression (Foci A, B, and C) manifested obvious recurrences, suggesting that HOXD‐AS2 might play an important role in chemo‐resistance.

### 3.7 HOXD‐AS2 interacts with IGF2BP2 to form an RNA‐protein complex

**FIGURE 4:**
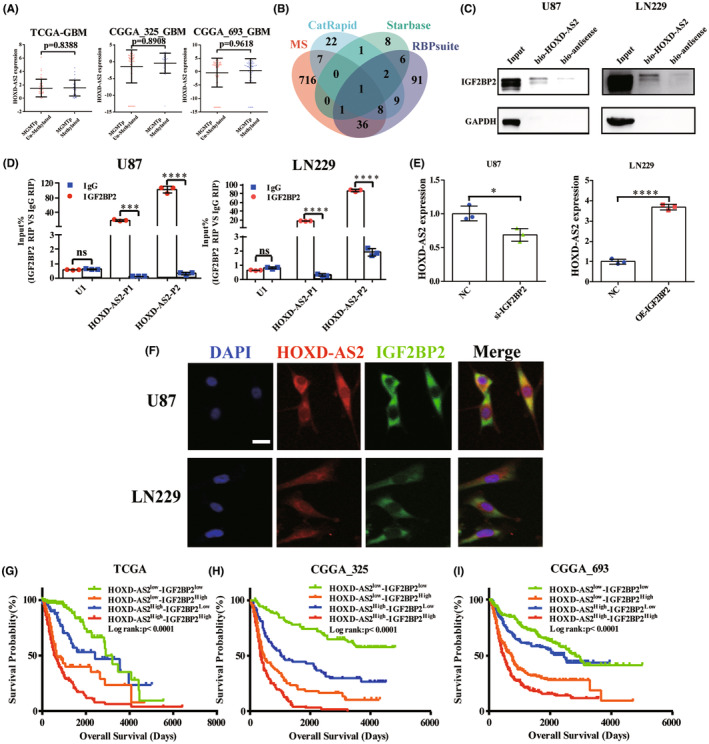
HOXD‐AS2 interacts with IGF2BP2 to form a RNA‐protein complex. (A) The HOXD‐AS2 expression in MGMT promoter un‐methylated/methylated GBM in TCGA and CGGA databases; (B) Online database prediction and mass spectrometry analysis identified proteins that HOXD‐AS2 might interact with; The intersected gene of all gene sets was IGF2BP2; (C) RNA‐protein pull‐down assay determined that HOXD‐AS2 interacted with protein IGF2BP2 in GBM cells; (D) RIP assays confirmed that HOXD‐AS2 interacted with protein IGF2BP2 in GBM cells; (E) The HOXD‐AS2 mRNA level after IGF2BP2 knockdown/over‐expression in GBM cells was quantified by RT‐qPCR; (F) The co‐localization of HOXD‐AS2 and protein IGF2BP2 was confirmed by FISH‐immunofluorescence double labeling assays; (G–I) Kaplan–Meier survival curves were used to examine the relationship of HOXD‐AS2/IGF2BP2 co‐expression status and overall survival of glioma patients in TCGA and CGGA databases; The experiment was repeated three times; Mann–Whitney test (A) and Student's *t*‐test for group comparisons; Log rank test for survival analysis; The asterisks denote significance (*p < 0.05, **p < 0.01, and ***p < 0.005), scale bar = 20 μm.

MGMT promoter methylation is associated with loss of MGMT expression that compromises DNA repair, leading to a favorable response to TMZ therapy.^30^ And MGMT promoter methylation status is an independent favorable prognostic factor in glioblastoma.^31^ We analyzed the association of HOXD‐AS2 and MGMT promoter methylation of glioblastoma in TCGA and CGGA databases and no significant difference was observed between methylated and un‐methylated glioblastoma (*p* = 0.8388, 0.8908, 0.9618, respectively, Figure 4A).

We sought to delineate the molecular mechanism of cytoplasmic HOXD‐AS2 independent of MGMT. Cytoplasmic lncRNA can always act as competitive endogenous RNAs to target miRNAs and proteins, affecting stability of target mRNAs and regulating the translation processes.^32^ LncRNAs are always exported to cytoplasm and interact with diverse RNA‐binding proteins,^33^ thus distributing persistently in cytoplasm and affecting cancer progression. From this perspective, we aimed to find RNA‐binding proteins which HOXD‐AS2 interacted to elaborate its mechanism. We integrated StarBase, RBPsuite, CatRapid online prediction results and mass spectrometry analysis and eventually identified IGF2BP2 (insulin‐like growth factor 2 mRNA‐binding protein 2), which manifested potentials to interact with HOXD‐AS2 (Figure 4B). Further RNA‐protein pull‐down and RIP assays verified that HOXD‐AS2 could interact with protein IGF2BP2 (Figure 4C,D); Meanwhile, RT‐qPCR assays revealed that IGF2BP2 could increase the HOXD‐AS2 expression (Figure 4E); FISH‐immunofluorescence double labeling assays also confirmed the co‐localization of HOXD‐AS2 and protein IGF2BP2 in cytoplasm (Figure 4F). Our results indicated that HOXD‐AS2 could interact with IGF2BP2 to form an RNA‐protein complex to exert its molecular function.

### 3.8 Elevated HOXD‐AS2/IGF2BP2 associates with worse prognosis of glioma

Accumulating evidence confirmed that IGF2BP2 links with various carcinomas including breast,^34^ ovarian,^35^ colon,^36^ and glioma.^37^ Previous results revealed that IGF2BP2 bound with HOXD‐AS2 and affected its expression, implying that whether HOXD‐AS2/IGF2BP2 genes' highly expression aggravated glioma progression. The survival analysis verified our hypothesis and revealed that with higher HOXD‐AS2/IGF2BP2 expression, patients experienced worse overall survival, on the contrary, patients with lower HOXD‐AS2/IGF2BP2 expression received survival benefit (log rank test, *p* < 0.0001, Figure 4F–H). These results implied that HOXD‐AS2/IGF2BP2 complex might play roles in glioma progression.

### 3.9 HOXD‐AS2/IGF2BP2 modulates STAT3 signaling and anti‐apoptosis activity

**FIGURE 5:**
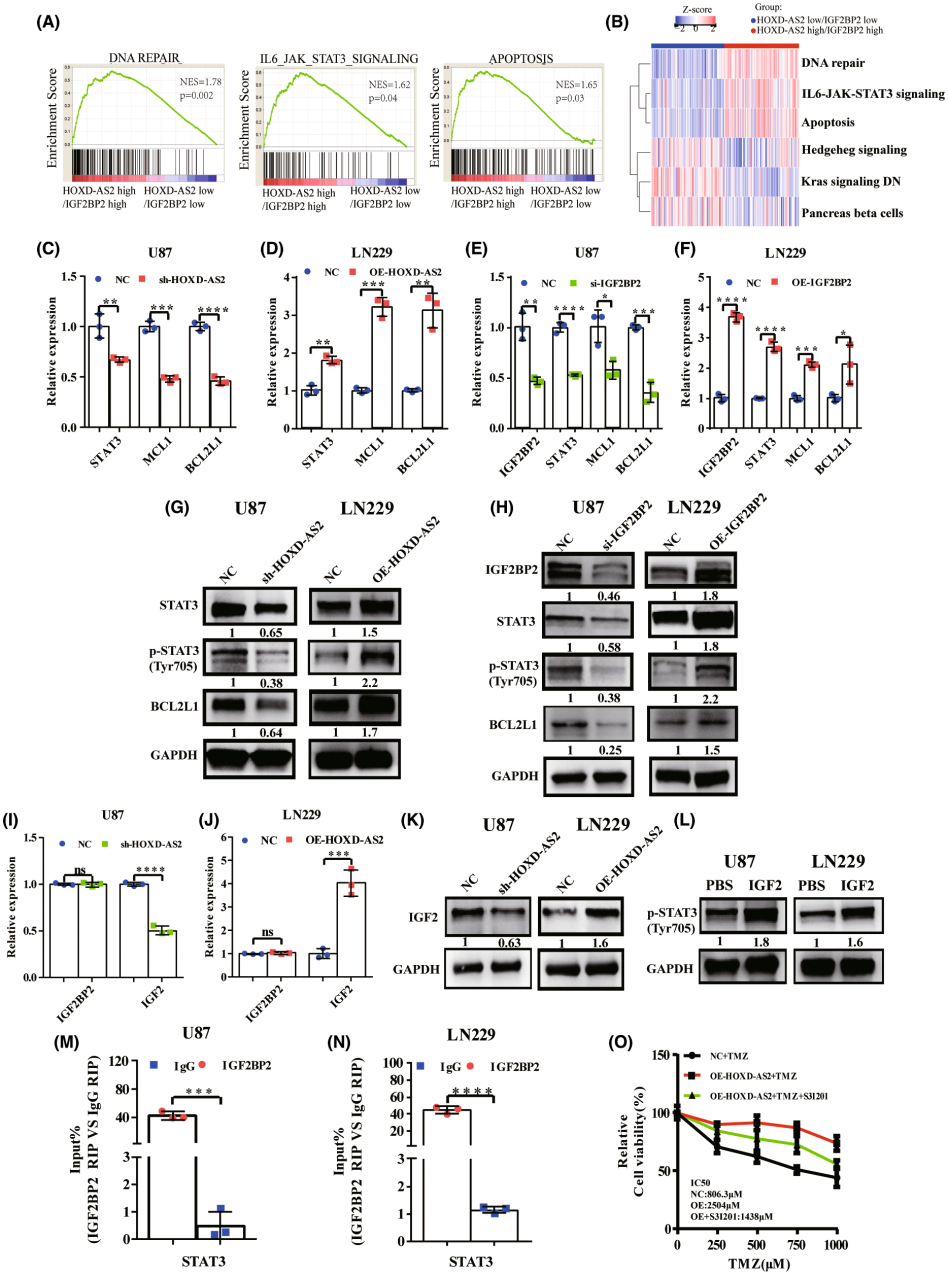
HOXD‐AS2/IGF2BP2 modulates STAT3 signaling pathway. (A) GSEA analysis revealed that high HOXD‐AS2/IGF2BP2 significantly enriched in DNA repair, apoptosis, and IL6‐JAK‐STAT3 signaling processes; (B) GSVA analysis revealed that high HOXD‐AS2/IGF2BP2 positively correlated with DNA repair, apoptosis and IL6‐JAK‐STAT3 signaling; (C, D) The mRNA levels of STAT3, MCL1, and BCL2L1 in GBM cells after HOXD‐AS2 knockdown/over‐expression were quantified by RT‐qPCR; (E, F) The mRNA levels of IGF2BP2, STAT3, MCL1, and BCL2L1 in GBM cells after IGF2BP2 knockdown/over‐expression were quantified by RT‐qPCR; (G) STAT3, STAT3 (phospho Tyr705), and BCL2L1 protein levels in GBM cells after HOXD‐AS2 knockdown/over‐expression were detected by immunoblotting; (H) IGF2BP2, STAT3, STAT3 (phospho Tyr705), and BCL2L1 protein levels in GBM cells after IGF2BP2 knockdown/over‐expression were detected by immunoblotting; (I, J) IGF2BP2 and IGF2 mRNA levels in GBM cells after HOXD‐AS2 knockdown/over‐expression were quantified by RT‐qPCR; (K) IGF2 protein level in GBM cells after HOXD‐AS2 knockdown/over‐expression was detected by immunoblotting; (L) STAT3 (phospho Tyr705) protein level after IGF2 recombinant protein treatment was detected by immunoblotting; (M, N) The interaction of IGF2BP2 and STAT3 mRNA was confirmed by RIP assays in U87 and LN229 cells; (O) Cells of different groups were treated with TMZ at different concentrations for consecutive 72 h, the relative cell viability was assessed immediately by CCK8 assays; The experiment was repeated three times; Student's *t*‐test for group comparisons; The asterisks denote significance (*p < 0.05, **p < 0.01, and ***p < 0.005), scale bar = 20 μm.

Subsequently, we discriminated all glioma samples into two groups of high and low HOXD‐AS2/IGF2BP2 expression in CGGA database, then GSEA and GSVA analysis were conducted to identify functional significance between groups. GSEA and GSVA analysis consistently revealed that high HOXD‐AS2/IGF2BP2 was positively correlated with functions of DNA repair, apoptosis, and STAT3 signaling (Figure 5A,B); We hypothesized whether HOXD‐AS2/IGF2BP2 modulated STAT3 signaling pathway and in vitro experiments were conducted to verify our prediction.

The RT‐qPCR assays showed that HOXD‐AS2 silencing not only suppressed mRNA level of STAT3, but also BCL2L1 and MCL1 (Figure 5C), which belong to the BCL2 family and are known as the downstream STAT3 target genes exerting anti‐apoptotic function; Conversely, HOXD‐AS2 over‐expression also increased their mRNA levels (Figure 5D). These results indicated that HOXD‐AS2 promoted the STAT3 signaling and enhanced BCL2 family gene transcription for anti‐apoptotic activity. Meanwhile, immunobloting assays also showed that HOXD‐AS2 promoted STAT3 protein expression and the phosphorylation at Tyr 705 residue, elevating protein level of downstream STAT3 target gene that regulated cellular anti‐apoptosis activity (Figure 5G). Similarly, knocking down IGF2BP2 significantly reduced mRNA levels of STAT3, BCL2L1, and MCL1 (Figure 5E,F), and the STAT3, BCL2L1 protein expression and phosphorylation level at Tyr 705 residue of STAT3 also decreased remarkably (Figure 5H). Our results indicated that HOXD‐AS2/IGF2BP2 modulates STAT3 signaling and anti‐apoptosis activity.

### 3.10 HOXD‐AS2/IGF2BP2 upregulates STAT3 signaling through promoting IGF2 and STAT3

IGF2BP2 was originally identified as a RNA‐binding protein capable of binding IGF2 mRNA and other transcripts.^38^ IGF2 acts as a secreted ligand, binding and activating IGF1 receptor and its downstream effector^39^ to exert its function, also involving in drug resistance of cancers.^40^ We found that IGF2 expression was promoted by HOXD‐AS2 (Figure 5I–K), whereas, HOXD‐AS2 did not affect the mRNA level of IGF2BP2 (Figure 5I,J), indicating that IGF2 expression regulated by IGF2BP2 depended on HOXD‐AS2 and HOXD‐AS2 cooperated with protein IGF2BP2 to elevate the IGF2 expression. Also, the IGF2 recombinant protein was then utilized to treat cells and we found that IGF2 promoted the STAT3 phosphorylation at Tyr 705 residue (Figure 5L), indicating that IGF2 activated the STAT3 signaling. These results revealed that HOXD‐AS2/IGF2BP2 elevated IGF2 expression cooperatively and then activated the STAT3 signaling.

On the other hand, previous results also showed that STAT3 mRNA and protein levels were also decreased after HOXD‐AS2/IGF2BP2 knocking down (Figure 5C–H). Thus, we speculated whether HOXD‐AS2/IGF2BP2 could also bind with STAT3 mRNA, forming a complex and then upregulating STAT3 expression, which was analogous to the IGF2 patterns. The RIP assays confirmed that IGF2BP2 could also bind with STAT3 mRNA (Figure 5M,N). These results indicated that up‐regulation of IGF2 and STAT3 was depended on HOXD‐AS2. Above all, our results demonstrated that HOXD‐AS2/IGF2BP2 complex increased the IGF2 and STAT3 expression to upregulate STAT3 signaling, simultaneously, the upregulated IGF2 also promoted the STAT3 signaling activation.

### 3.11 STAT3 inhibition reversely sensitizes GBM to TMZ in HOXD‐AS2 over‐expression condition

Previous results revealed the role of HOXD‐AS2 in modulating temozolomide sensitivity through promoting STAT3 signaling and BCL2 anti‐apoptotic genes expression. Subsequently, the cell viability assay was performed to verify the importance of HOXD‐AS‐STAT3 axis in TMZ sensitivity and showed that HOXD‐AS2 promoted the TMZ resistance, the IC50 value increased to 2504 μM from 806.3 μM, nevertheless, STAT3 inhibition reversely sensitized glioblastoma to temozolomide, and IC50 value decreased to 1438 μM from 2504 μM (Figure 5O), indicating the importance of HOXD‐AS2‐STAT3 axis in TMZ‐sensitivity regulation.

### 3.12 STAT3 elevates HOXD‐AS2 expression through transcription activation

**FIGURE 6:**
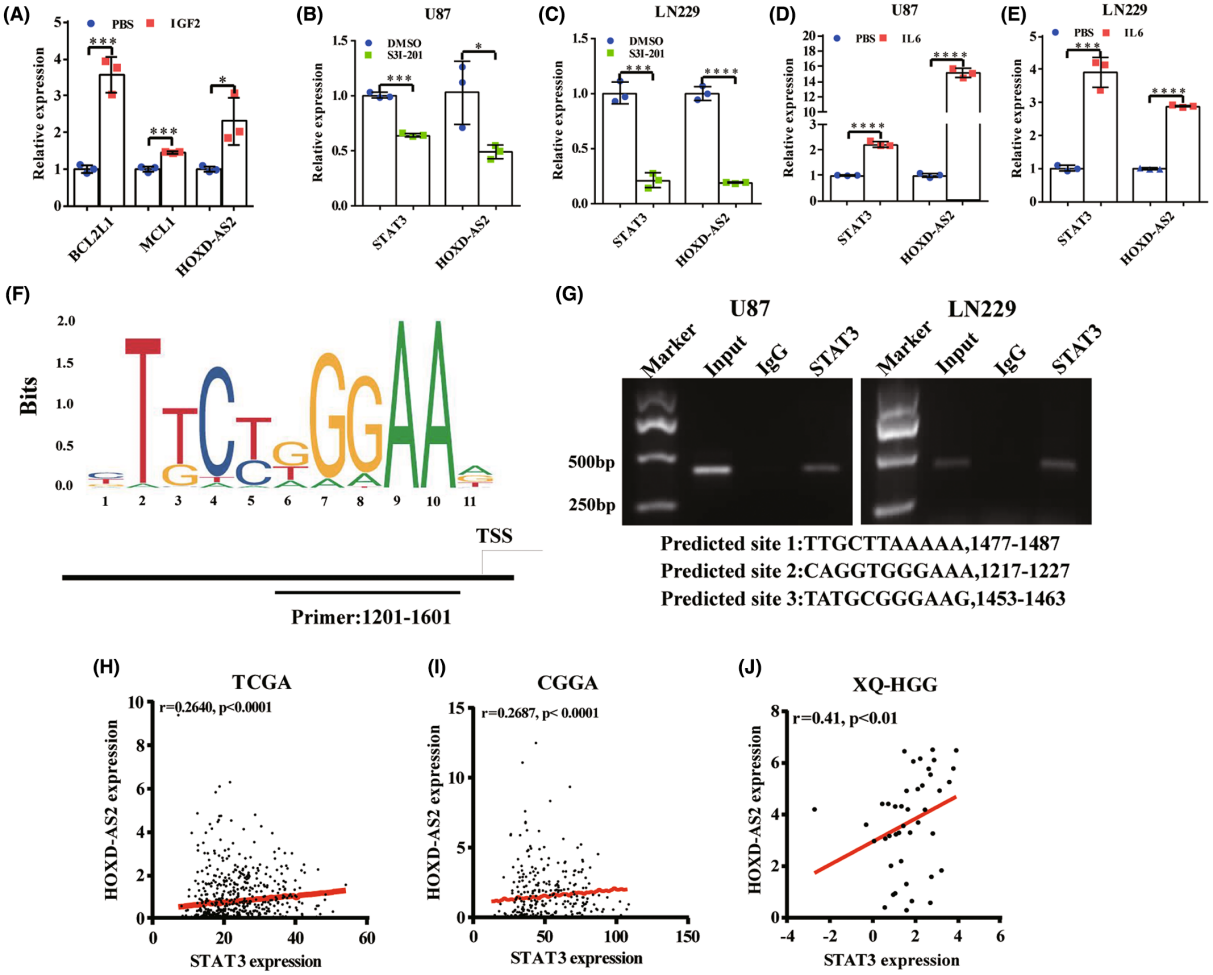
STAT3 elevated HOXD‐AS2 expression through transcription activity. (A) MCL1, BCL2L1, and HOXD‐AS2 mRNA levels after IGF2 recombinant protein treatment (1 μg/mL, 72 h) in GBM cells were quantified by RT‐qPCR; (B, C) STAT3 and HOXD‐AS2 mRNA levels in U87 and LN229 cells after S3I‐201 treatment (50 μM, 48 h) and (D, E) IL6 treatment (200 ng/mL, 72 h) were quantified by RT‐qPCR; (F) The predicted binding motif sequence of STAT3 on HOXD‐AS2 promoter region; (G) The binding of STAT3 and HOXD‐AS2 promoter region was confirmed by ChIP‐PCR assays; (H–J) HOXD‐AS2 was positively correlated with STAT3 expression in TCGA, CGGA, and XQ‐HGG cohorts (Spearman's coefficient); The experiment was repeated three times; Student's *t*‐test for group comparisons; The asterisks denote significance (*p < 0.05, **p < 0.01, and ***p < 0.005), scale bar = 20 μm.

Previous results showed that IGF2 could promote STAT3 phosphorylation and further RT‐qPCR assays showed that mRNA levels of BCL2L1 and MCL1 were also increased after IGF2 recombiant protein treatment, indicating the activation of STAT3 signaling and anti‐apoptosis activity (Figure 6A); Additionally, the elevation of HOXD‐AS2 level was also observed and it attracted our interest that whether the STAT3 signaling could affect HOXD‐AS2 expression inversely, thus forming a positive feedback loop. A selective STAT3 inhibitor, S3I‐201 and a typical STAT3 activator, IL6 were utilized to treat cells to simulate STAT3 inhibition and activation. The RT‐qPCR assays revealed that the HOXD‐AS2 expression was changed correspondingly after treatments, indicating that STAT3 could promote HOXD‐AS2 expression (Figure 6B–E). Additionally, the gene co‐expression analysis also showed that HOXD‐AS2 was positively correlated with the STAT3 expression (Figure 6H–J).

STAT3 is a canonical transcription factor and persistent STAT3 activation affects cellular processes and target genes' transcriptions in gliomas. We hypothesized that STAT3 promoted HOXD‐AS2 expression by exerting its function as a transcription factor. To verify our hypothesis, the JASPAR database was utilized to predict whether there existed potential binding sites of STAT3 on HOXD‐AS2 promoter region. The potential binding sequence is shown in Figure 6F, and we predicted a portion of promoter region between −1601/−1201 bp nucleotides from the transcription starting site (TSS), consisting of three putative binding sites, located at −1487/−1477 (site 1), −1227/−1217 (site 2), and −1463/−1453 (site 3) from the TSS. The ChIP‐PCR assays confirmed that STAT3 could bind with the predicted HOXD‐AS2 promoter region (Figure 6G). These results revealed that STAT3 elevated HOXD‐AS2 expression through transcription activation.

## 4 DISCUSSION

Despite the success of temozolomide, chemo‐resistance due to the highly heterogeneity of glioblastoma affects patients' prognosis remarkably. LncRNAs are the key regulators of diverse malignant behaviors of glioma including therapeutic resistance.^41^ Our bio‐informatic analysis screened out and identified the up‐regulation of HOX‐related lncRNAs in glioblastoma, which play critical roles in gene regulation and chromatin dynamics of cancers.^42^ Considering that HOXD‐AS2 was the most upregulated member among them and growing studies revealed its implications in glioma, it deserves an investigation in depth.

First, we validated the highly HOXD‐AS2 expression in high‐grade gliomas we collected in clinic. Furthermore, we validated and indicated that elevated HOXD‐AS2 associated with poor prognosis in gliomas of different pathological grades and it was promising to be a prognostic factor.

We subsequently investigated and explored the potential functions of HOXD‐AS2 in glioma. TMZ‐induced DNA damage and cell death are accompanied by DNA repair system activation, and our analysis revealed that (i) HOXD‐AS2‐related genes were mainly located in nucleus; (ii) HOXD‐AS2 was mainly involved in the DNA damage repair processes. These results implied that HOXD‐AS2 was associated with the TMZ‐induced therapeutic response. We also found that patients with low HOXD‐AS2 expression achieved better survival after TMZ treatment through survival analysis, implying its underlying function in TMZ sensitivity regulation.

In vitro assays showed that HOXD‐AS2 promoted the TMZ resistance of GBM cells; In vivo animal experiments demonstrated that combination of TMZ and HOXD‐AS2 silencing induced the remarkable DNA damage activity, exhibiting more satisfactory therapeutic effects, simultaneously, the tumor growth was also inhibited significantly. Meanwhile, a special clinical case of multiple GBM that a patient with four intracranial GBM foci attracted our interest and attention. We found that the foci of lowest HOXD‐AS2 expression manifested no obvious signs of recurrence after standard treatment. This case provided evidence of relationship between HOXD‐AS2 and TMZ resistance. We will collect more clinical samples to confirm this association in future cohort study.

Mechanically, despite MGMT plays dominant role in temozolomide resistance^43^ due to its ability of removing O^6^‐alkylguanine DNA adducts, accumulating studies revealed that MGMT may not be the unique factor. Signaling pathway dysfunction, diverse DNA repair systems, autophagy, epigenetic modifications, microRNAs, and extracellular vesicle production may contribute to the TMZ resistance independent of MGMT.^44^ Although it has been reported HOXD‐AS2 promoted MGMT expression,^17^ considering the high HOXD‐AS2 but low MGMT expression in U87 and LN229 cells,^45^, ^46^ we speculated that HOXD‐AS2‐mediated TMZ resistance was mechanically independent on MGMT. Meanwhile, the association of HOXD‐AS2 and MGMT promoter methylation status was analyzed and no significant differences of HOXD‐AS2 expression between un‐methylated and methylated GBMs.

We further found that HOXD‐AS2 interacted with IGF2BP2 to form a RNA‐protein complex and highly HOXD‐AS2/IGF2BP2 was involved in STAT3 signaling and anti‐apoptosis activities, leading to worse survival. IGF2BP2 belongs to the IGF2BP family which consists of three members (IGF2BP1, 2, 3), capable of associating with IGF2 and other transcripts and mediating their processing,^47^ additionally, it can also affect the chemo‐resistance of tumors.^48^ Aberrant STAT3 activation and highly phosphorylation positively correlate with the grades and poor prognosis of glioma,^49^ and are also involving in chemo‐resistance.^50^, ^51^ The phosphorylation at tyrosine‐705 (Y705) residue of C‐terminal is the dominant mechanism of STAT3 activation that two pSTAT3‐Y705 proteins associate to trans‐locate to nucleus and induce genes' transcription. Cells respond to DNA damage by activating complex signaling networks that decide cellular fate. Decision between cell survival and death following DNA damage rests on factors involved in apoptosis.^52^ In our study, we found that upregulated HOXD‐AS2/IGF2BP2 promoted STAT3 signaling, phosphorylating STAT3 at tyrosine 705 residue and elevating expression of BCL2L1 and MCL1, which promoted cellular anti‐apoptotic activity. Furthermore, we found that HOXD‐AS2/IGF2BP2 elevated IGF2 and then it promoted the STAT3 signaling; Simultaneously, we also found that IGF2BP2 interacted with STAT3 mRNA and elevate its expression for STAT3 activation. These results indicated that HOXD‐AS2/IGF2BP2 promoted the STAT3 signaling and anti‐apoptotic activity to regulate TMZ sensitivity.

Eventually, we focused on whether STAT3 affected HOXD‐AS2 expression inversely. We applied the selective STAT3 inhibitor, S3I‐201, which functions via inhibition of STAT3 dimerization,^53^ and IL6, the most well‐known activator of STAT3^54^ to inhibit and stimulate STAT3 signaling and found that STAT3 activation promoted the HOXD‐AS2 expression. Considering that STAT3 is a canonical transcription factor and accumulating evidences also confirmed that transcription activation of lncRNAs plays roles in tumorigenesis.^55^ We hypothesized that HOXD‐AS2 might be transcriptionally regulated by STAT3 and our results confirmed that STAT3 could bind with HOXD‐AS2 promoter region. This mechanism could probably explain the highly HOXD‐AS2 expression status in GBM, which is not clearly identified and clarified yet.

Overall, we described a HOXD‐AS2/STAT3 positive feedback loop in GBM that STAT3‐induced lncRNA HOXD‐AS2 interacted with protein IGF2BP2 to form a complex, upregulating IGF2 and STAT3 signaling and promoting the BCL2 anti‐apoptotic genes' expression, thus attenuating temozolomide sensitivity. However, this work also has some limitations: (i) The concrete binding regions of STAT3 and HOXD‐AS2 promoter within these predicted sites should be further determined; (ii) In consideration of the post‐transcriptional regulation patterns of cytoplasmic HOXD‐AS2, concrete molecular mechanism of HOXD‐AS2/IGF2BP2 complex modulating IGF2 and STAT3 should be elucidated in depth; (iii) We concentrated on the effect of HOXD‐AS2‐STAT3 feedback in regulating TMZ resistance; IGF2 act as an effector of HOXD‐AS2 which indirectly promotes STAT3 signaling, the role of IGF2 in TMZ resistance regulation, and whether IGF2 silencing could reverse the TMZ resistance induced by HOXD‐AS2 deserves further investigation.

## 5 CONCLUSION

In summary, our study described a HOXD‐AS2‐STAT3 positive feedback loop which attenuates TMZ sensitivity in glioblastoma and it revealed clinical implications involving HOXD‐AS2 and TMZ sensitivity. HOXD‐AS2 is a potential prognostic biomarker in glioma and whether exploring this feedback loop will provide a novel therapeutic candidate deserves further expectations.

## AUTHOR CONTRIBUTIONS

Zuo‐Xin Zhang and Sheng‐Qing Lv designed this study and wrote the manuscript; Zuo‐Xin Zhang, Peng Ren, Yong‐Yong Cao, Ting‐Ting Wang, Guo‐Hao Huang, Yao Li, Shuo Zhou, Wei Yang, Lin Yang, Guo‐Long Liu, Yan Xiang, Yu‐Chun Pei, and Qiu‐Zi Chen carried out the experiments; Zuo‐Xin Zhang performed the data analysis; and Ju‐Xiang Chen helped in revising the manuscript.

## CONFLICT OF INTEREST STATEMENT

All authors declared that they have no competing interests.

## Supporting information


Table S1

Table S2

File S1

## Data Availability

RNA‐seq data of glioma samples analyzed in this work can be acquired from the Cancer Genome Atlas (TCGA) dataset (https://portal.gdc.cancer.gov/), and the Chinese Glioma Genome Atlas (CGGA) dataset (http://www.cgga.org.cn).
