# Supplementary material for: HOXD‐AS2‐STAT3 feedback loop attenuates sensitivity to temozolomide in glioblastoma
Source: CNS Neurosci Ther. 2023 Jun 12;29(11):3430–45. doi: 10.1111/cns.14277 (PMC10580348; doi:10.1111/cns.14277)

Full unedited blot for Figure 4C

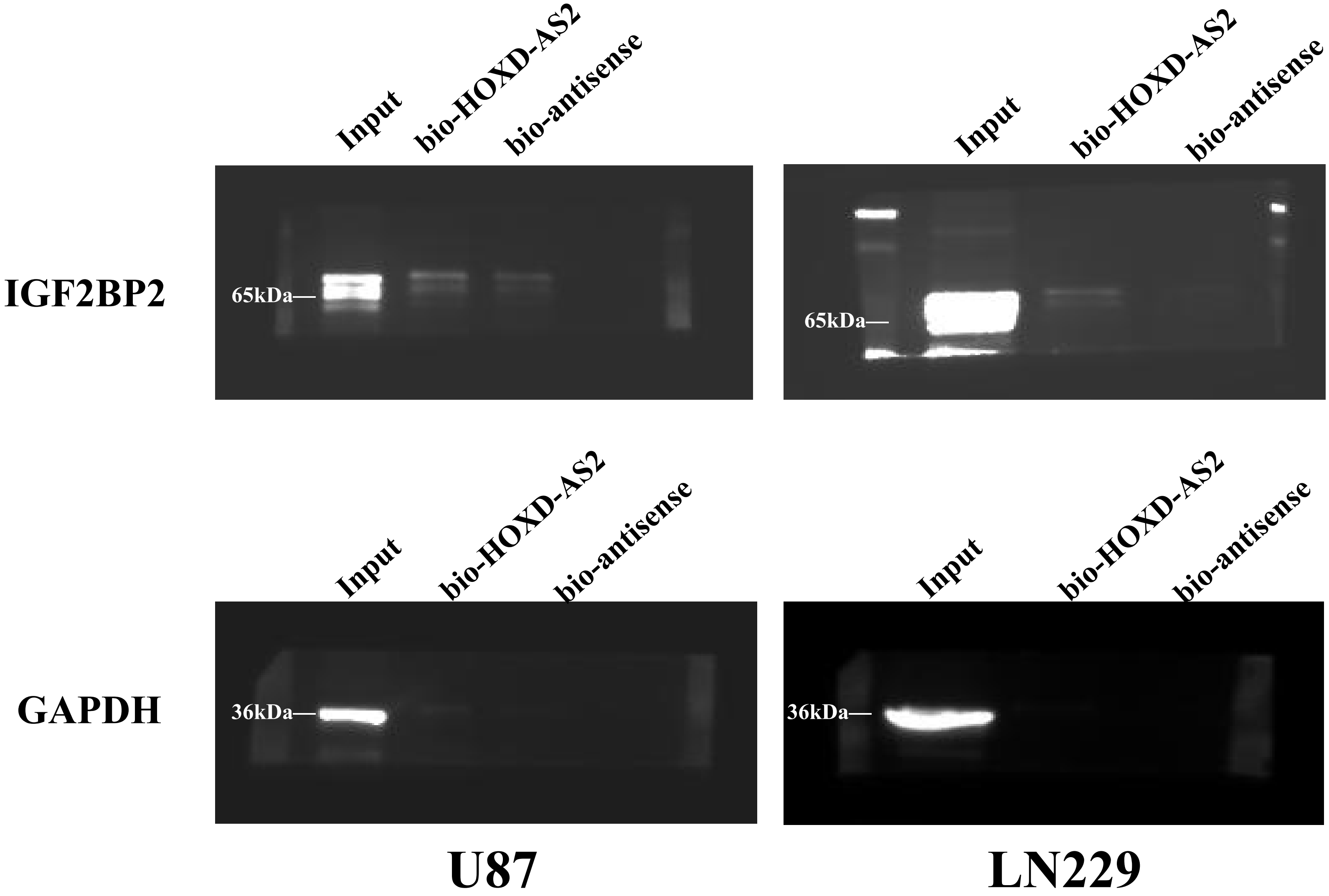

Full unedited blot for Figure 5G

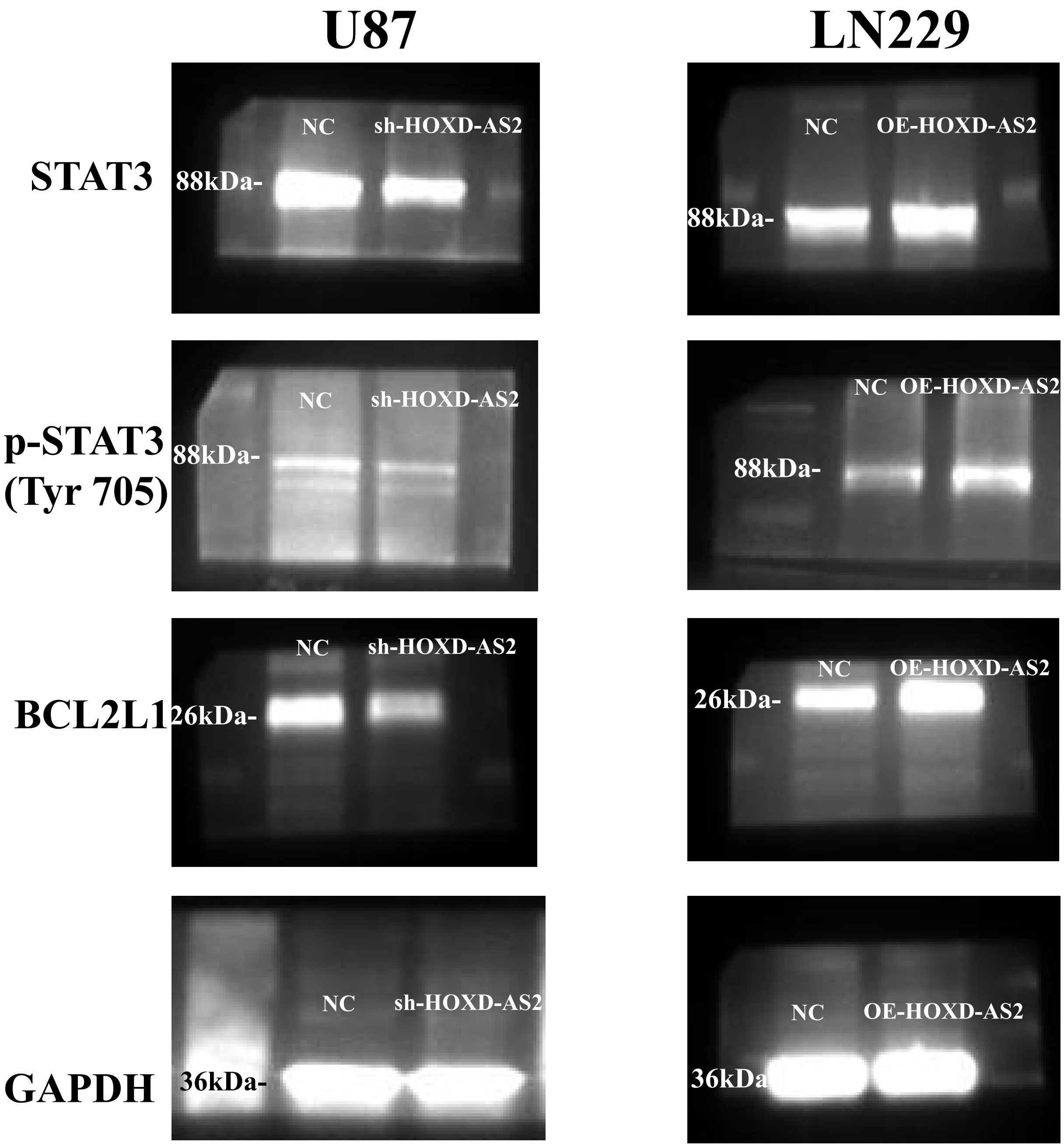

Full unedited blot for Figure 5H

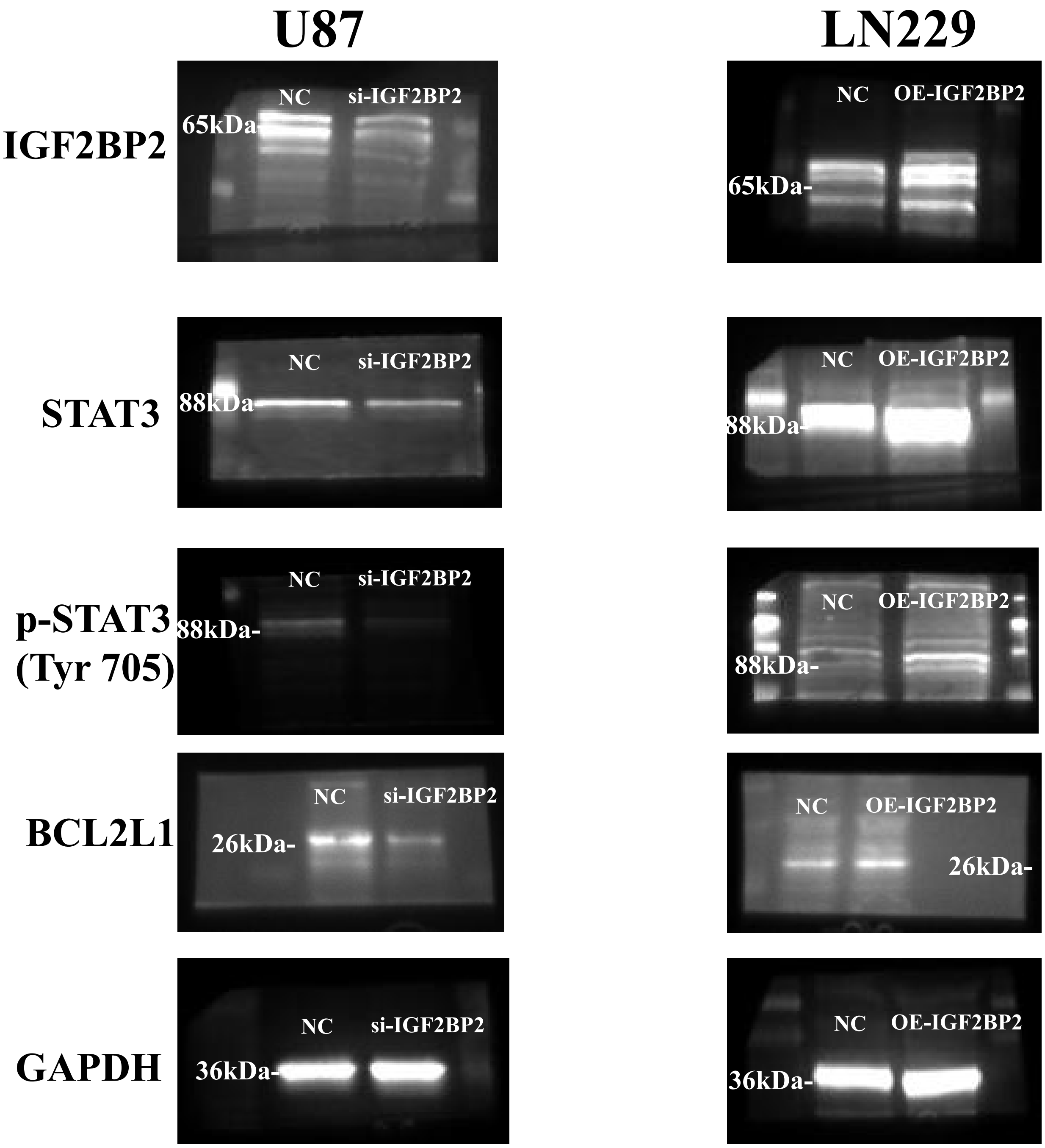

Full unedited blot for Figure 5K

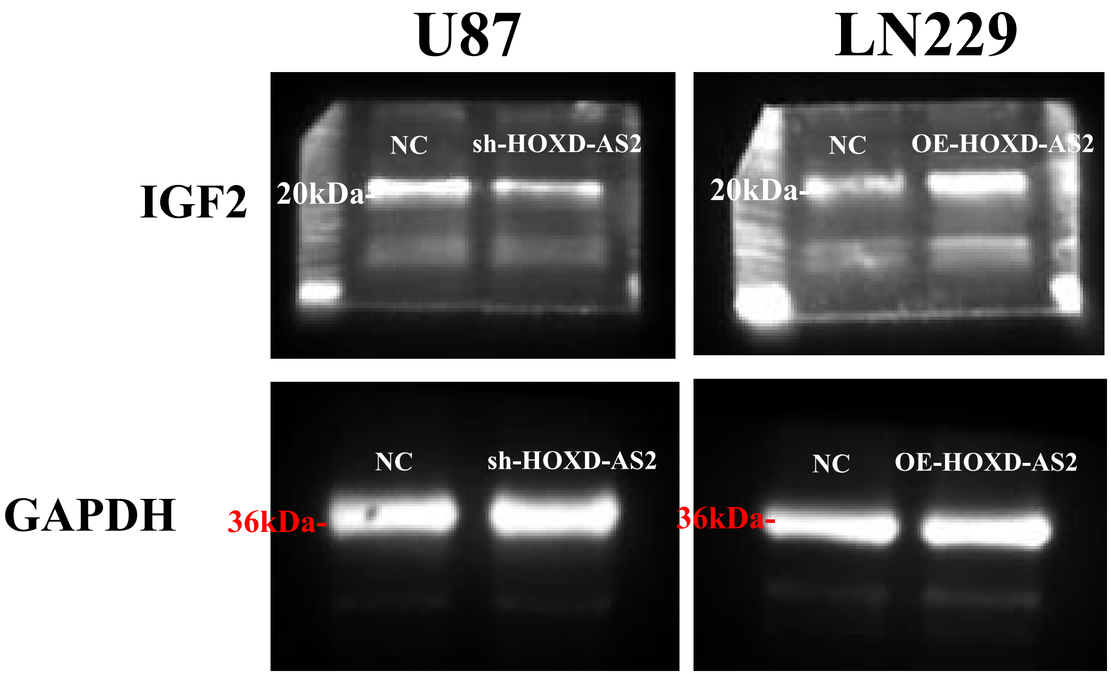

Full unedited blot for Figure 5L

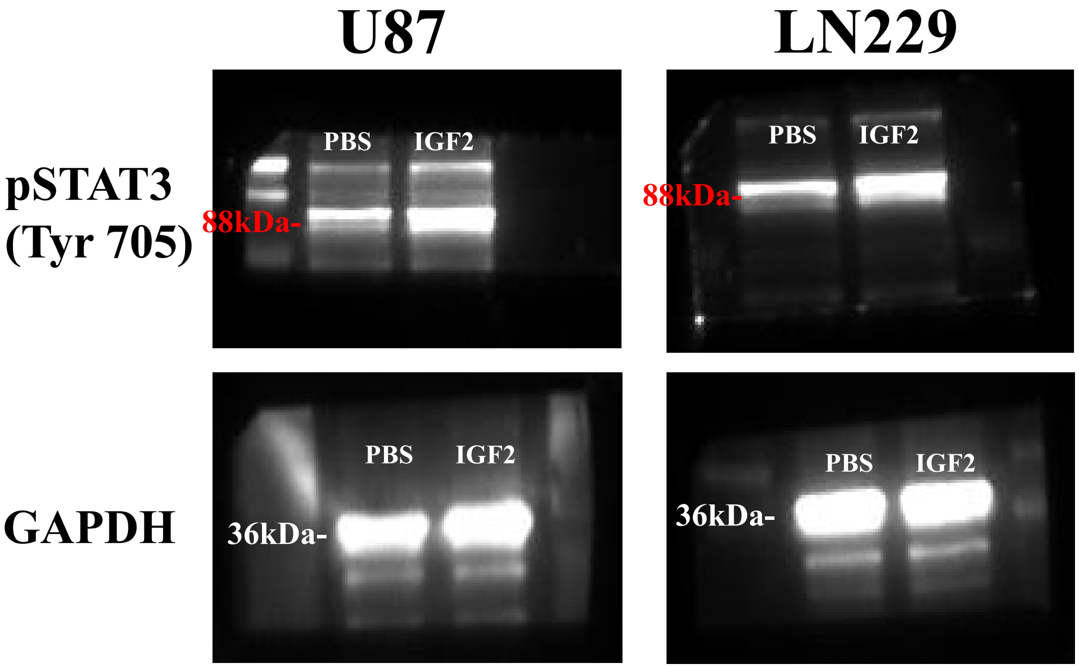

Full unedited blot for Figure 6G

U87

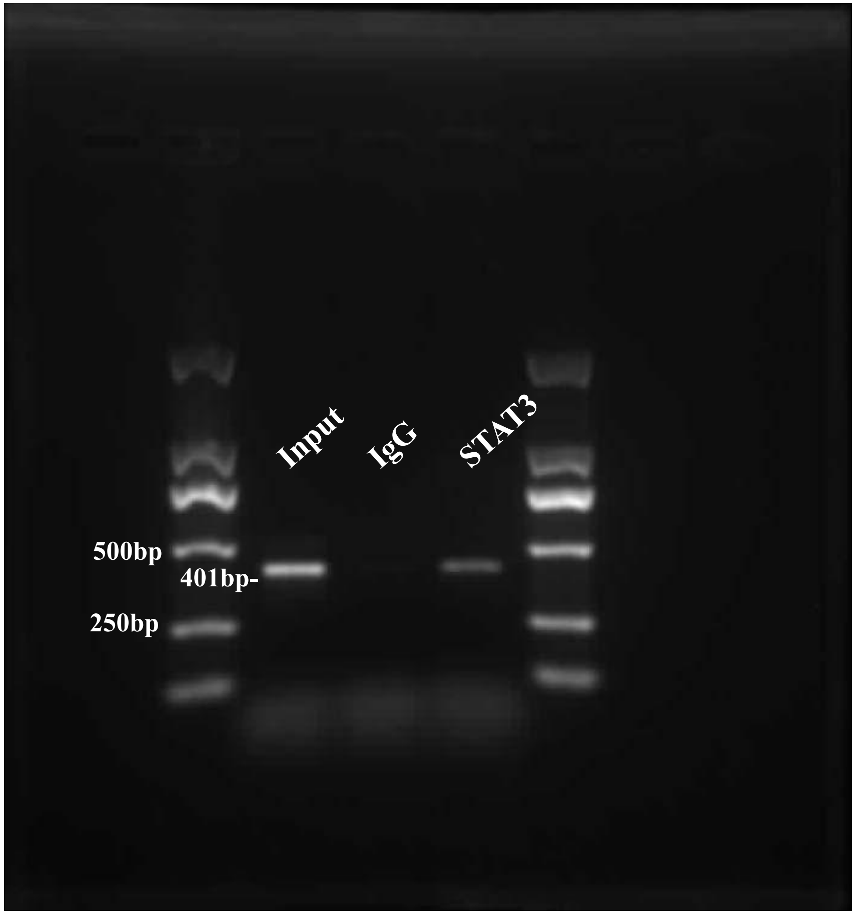

LN229

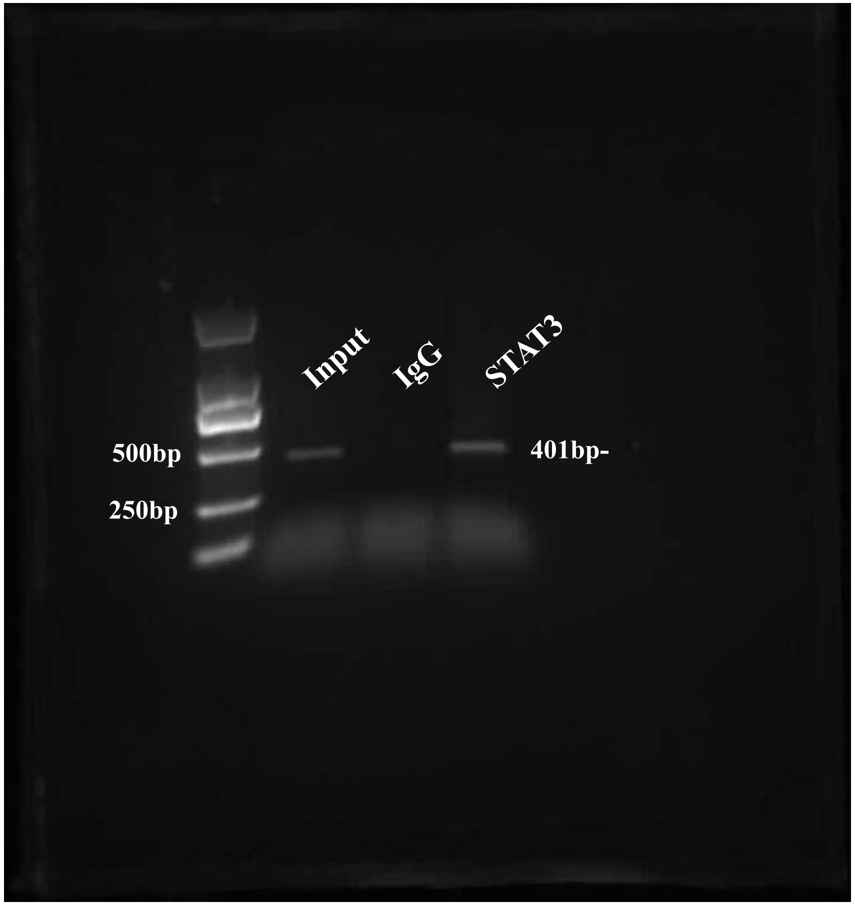

Supplement: Supplementary file 3 — File S1 [file CNS-29-3430-s001.pdf]
